# Structure of the Lung

**Published:** 1842-11-26

**Authors:** 


*Structure of the Lung.*—At the Academy of Sciences, Paris, on the 19th September, 1842, M. Bourgery read a memoir on the relation which exists between the structure of the lung and its functional capacity in both sexes and at different periods of life.

The author first gives the measurements of the minute pulmonary apparatus in the adult, and then passes to the microscopic examination of its texture at different periods of life. He shows that the developments of the æriel and sanguineous capacities of the pulmonary apparatus is much influenced by age, appearing to be in inverse proportion at the two extremes of life.

In infancy the vascularity and æriel capacity of the lung are very great, and this, perhaps, may occasion the extreme plasticity of the blood at this period.

The great energy of the respiration, arising from the full but equal development of the sanguineous and æriel systems, is characteristic of adolescence, and manifests itself by the rich qualities of the blood peculiar to puberty.

In the adult the respiratory apparatus remains stationary for some time, but as years go on the air-cells partially give way, and the blood-vessels become obliterated, and old age ensues with its feeble and impoverished circulation.

From these facts the author thinks that, in a general point of view, man, at his different periods of life, presents an analogy to the two classes of vertebrate animals in which the extremes of the respiratory functions are observed.

As he approaches towards puberty the lung is gradually developed, and offering every year larger and larger surfaces to the air, gives rise to a function similar to that of the bird. In old age, on the contrary, the lung is gradually broken up into air cavities of increasing magnitude, while the circulation is diminished in the same proportion; and thus the respiration, both in its real capacity and in the structural changes of the organ effecting it, assumes the characters peculiar to reptiles.

